# A machine learning driven automated system for safety data sheet indexing

**DOI:** 10.1038/s41598-024-55231-1

**Published:** 2024-02-22

**Authors:** Aatish Suman, Misbah Khan, Veeru Talreja, Julia Penfield, Stephanie Crowell

**Affiliations:** Velocity EHS Inc, Chicago, IL 60654 USA

**Keywords:** Environmental impact, Environmental impact, Information technology, Computational science

## Abstract

Safety Data Sheets (SDS) are foundational to chemical management systems and are used in a wide variety of applications such as green chemistry, industrial hygiene, and regulatory compliance, among others within the Environment, Health, and Safety (EHS) and the Environment, Social, and Governance (ESG) domains. Companies usually prefer to have key pieces of information extracted from these datasheets and stored in an easy to access structured repository. This process is referred to as SDS “indexing”. Historically, SDS indexing has always been done manually, which is labor-intensive, time-consuming, and costly. In this paper, we present an automated system to index the composition information of chemical products from SDS documents using a multi-stage ensemble method with a combination of machine learning models and rule-based systems stacked one after the other. The system specifically indexes the ingredient names, their corresponding Chemical Abstracts Service (CAS) numbers, and weight percentages. It takes the SDS document in PDF format as the input and gives the list of ingredient names along with their corresponding CAS numbers and weight percentages in a tabular format as the output. The system achieves a precision of 0.93 at the document level when evaluated on 20,000 SDS documents annotated for this purpose.

## Introduction

The prominent rise of ESG over the last 3 years has generated a focus on enterprise-wide environmental practices^1^. The product compliance regulatory landscape has also become increasingly stringent^2^, driving chemical producers, distributors, and users to align their practices more with the principles of industrial hygiene and green chemistry. According to the US Environmental Protection Agency (EPA)^3^, green chemistry is the design of chemical products and processes that reduce or eliminate the use or generation of hazardous substances across the life cycle of a chemical product. Its goal is to reduce pollution at its source while eliminating hazards from products and improving energy efficiency. According to the American Industrial Hygiene Association^4^, there is also a direct overlap between these goals and those of industrial hygiene. A system to manage and access the properties and composition of the chemical products present on site is a crucial step in the practices of green chemistry and industrial hygiene.

This information is available in SDSs, and the Hazard Communication Standard (HCS) (29 CFR 1910.1200(g))^5^ by the Occupational Safety and Health Administration (OSHA) requires every chemical manufacturer, distributor, or importer to provide readily accessible SDSs for each hazardous chemical to their workers. Indexing (converting to a structured format) this information is not only essential for an efficient chemical management system, but also the first step in building compliance, monitoring, and hazard identification systems that can lead to better environmental practices and help companies align their goals with those of green chemistry.

**Figure 1:**
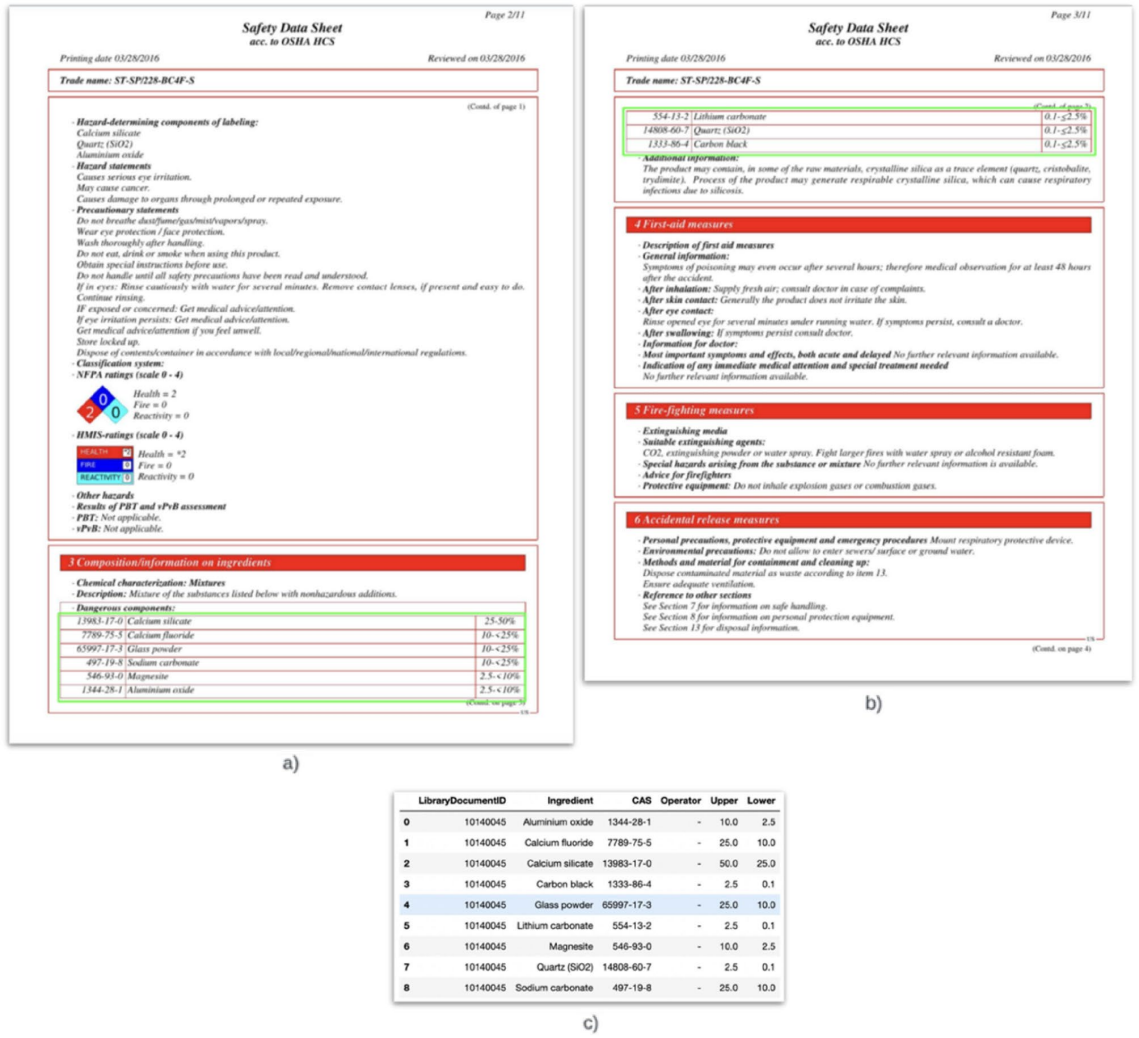
(**a**,**b**) Consecutive pages of an SDS document with the tables identified (outlined) in the composition section (split across the pages). (**c**) Final output for the document.

Historically, indexing has been done through labor-intensive manual work, which is time-consuming and expensive. In this paper, we describe our developed method for automated indexing of the composition information of a chemical product from an SDS document, with the results obtained on our datasets. Specifically, we focused on indexing the ingredient names, their corresponding Chemical Abstracts Service (CAS) numbers, and weight percentages. The solution receives the SDS as a PDF file as input and gives the list of ingredient names, along with their corresponding CAS numbers and weight percentages, in a 2D structure as the output, which can then be stored in a CSV file or a structured database (Fig. 1).

The rest of the paper is organized as follows—in Section “Related work”, we present a review of some of the existing methods for extracting information from documents; Section “Methodology and architecture” describes the architecture of the developed system; in Section “Results and discussion”, we present the results of the system on our datasets; and Section “Conclusion and future work” concludes the paper. The patent information is available in the *competing interests* section.

## Related work

The use of machine learning techniques to extract information from documents has recently become highly popular and led to a topic of research called *document AI* or *document understanding*. In the last decade, deep learning models have become the norm for document understanding tasks^6–8^. More recently, Graph Neural Networks have also been used for table detection^9^, as well as multimodal feature extraction using both text and image inputs for richer representation and contextualization^8,10,11^. Inspired by the pre-trained language models in Natural Language Processing (NLP), multimodal self-supervised pre-training has made rapid progress in this field. LayoutLM^12^ modified the BERT^13^ architecture by adding spatial coordinate embeddings, extending the masked language modeling task to masked visual-language modeling. LayoutLMv2^14^ further improved over it by treating the visual features as separate tokens and using two pre-training tasks, text-image matching, and text-image alignment, to allow cross-modal interaction. SelfDoc^15^ proposed a more coarse-grained contextualization over a block of content instead of individual words, while StructuralLM^16^ proposed cell-level position embeddings to utilize the interactions of cell and layout information. StrucTexT^17^ introduced a unified framework for efficiently extracting semantic features from different levels and modalities to handle the entity-labeling and entity-linking tasks simultaneously, while DocFormer^18^ designed a multimodal self-attention layer allowing the sharing of the learned spatial embeddings across both visual and textual modalities. LayoutLMv3^19^ proposed a linear Word-Patch Alignment objective to allow cross-modal interactions, and unified text and image masking objectives, achieving state-of-the-art results while reducing the number of parameters generally required for such multimodal document AI architectures.

Although these models have achieved great results on standard datasets for tasks like key information extraction, our experiments with one-shot methods for the objective of identifying each entity (name, CAS, and weight) individually, did not yield satisfactory results at the document level (although these methods were not multimodal). To the best of the authors’ knowledge, this is the first instance of an article describing an automated system for extracting composition information from an SDS.

## Methodology and architecture

**Figure 2:**
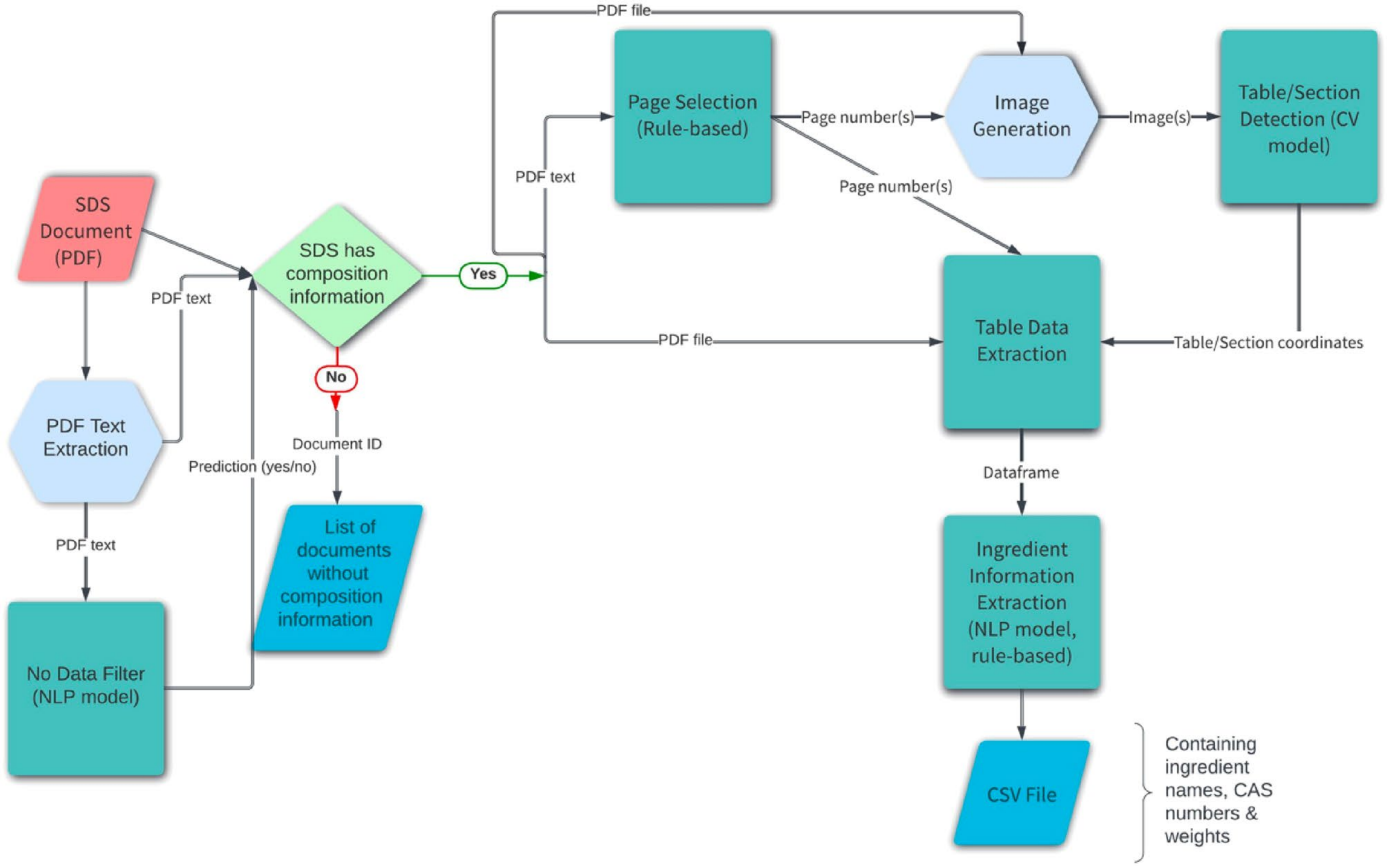
Model pipeline.

The failure of the one-shot methods (as described above) can be attributed to the variety in the structure and format of the SDS documents (across manufacturers, regions, etc.), making the development of a generalized automated system with high precision a challenging task. The difficulty of the specific problem of extracting composition information from the documents is further exacerbated by the fact that we need to extract multiple instances of three pieces of information (name, CAS, and weight) along with their associations with each other where the presence of two of them (CAS and weight) is not mandatory and the information can be split across multiple pages of the document and arranged row-wise or column-wise. Therefore, we decided to break the objective down to multiple simpler tasks. Figure 2 shows the steps of the pipeline used, the details of which are described in this section.

### Pipeline overview

The SDS documents generally follow the format described in the OSHA Brief DSG BR-3514^20^ where the document is divided into 16 sections. The composition information is usually present in Section 3 (or Section 2 in some older formats). In some cases where the chemical product is composed of a single ingredient, the composition section does not contain any additional information, and the product itself has a CAS number mentioned in Section 1. For such cases, the system outputs the product name as the ingredient name along with the CAS number. The ingredient information is usually arranged in a tabular format, and the system is designed to first identify the location of the tables. For the cases where no table is identified, the system contains an additional step for identifying the location of the composition section. It consists of the following stages (Fig. 2):

- PDF text extraction (preprocessing step)
- No data filter
- Page selection
- Image generation (preprocessing step)
- Table/Section detection
- Table data extraction
- Ingredient information extraction

Detailed descriptions of these steps are given below. The entire workflow was implemented in Python.

### PDF text extraction

The text from the PDF documents was extracted one page at a time using a combination of three PDF extraction tools—Apache PDFBox^21^ (executed through Python), pdfplumber^22^, and pdftotext^23^. The accuracy of each of these tools is not 100%, and it was observed that in certain cases, one of them would fail to produce any output for a document while another would succeed. It was also observed that Apache PDFBox produced the best results in general, and was therefore used first. If no output was produced, pdfplumber was tried next followed by pdftotext. A series of preprocessing steps, including removal of endline arguments, removal of trailing and leading white space, and conversion to lower case, were then applied to the text. Stop words such as conjunctions and prepositions were also removed using the Gensim^24^ library’s default set, as they do not add any value in the context of the Information extraction.

### No data filter

A small but significant percentage of SDS documents (6–8% in our datasets) do not contain any composition information. This generally occurs when the product and its ingredients are not hazardous. Although OSHA does not require employers to maintain SDSs for such products, many organizations often supply SDSs for liability purposes. Experiments showed superior overall performance of the pipeline if a separate filter was used to first identify such documents; this filter forms the first step of the pipeline.

A binary classifier, which received the preprocessed concatenated text of all the pages as the input and predicted whether the document contained any ingredient information, was trained. The model used for this purpose was a pre-trained Bidirectional Encoder Representations from Transformers (BERT) model, fine-tuned on a custom dataset (details in Supplementary Table S1). BERT is a transformer-based Large Language Model (LLM) designed for NLP applications. Its architecture consists of several transformer encoders stacked together. Each encoder consists of two sub-layers, a self-attention layer, and a feed-forward layer, and the model has been pre-trained on unlabeled data (about 3300 M words) extracted from BooksCorpus and Wikipedia. Additionally, it has been pre-trained in a bidirectional nature, which means that the model learns information from a sequence of words, not only from left to right, but also from right to left. The BERT model expects a sequence of tokens as an input, and it outputs an embedding vector of size 768 for each of the tokens. We can use these vectors as input for different kinds of NLP applications, such as text classification, next sentence prediction, named entity recognition, question-answering, etc.

The input text was split into tokens using the WordPiece Tokenizer used by BERT and grouped into chunks of size equal to the maximum sequence length used for the model (mentioned in the Supplementary Table S1). Each chunk along with its corresponding label was fed as a separate record to the model. For the documents with no ingredient information, the label for each of the chunks was the same (defined as the positive class). For the other class of documents, the label for each chunk was generated by checking whether the corresponding text contained any of the ingredient information or not. The prediction for the whole document was made by checking whether any of its chunks contained the ingredient information or not.

### Page selection

**Figure 3:**
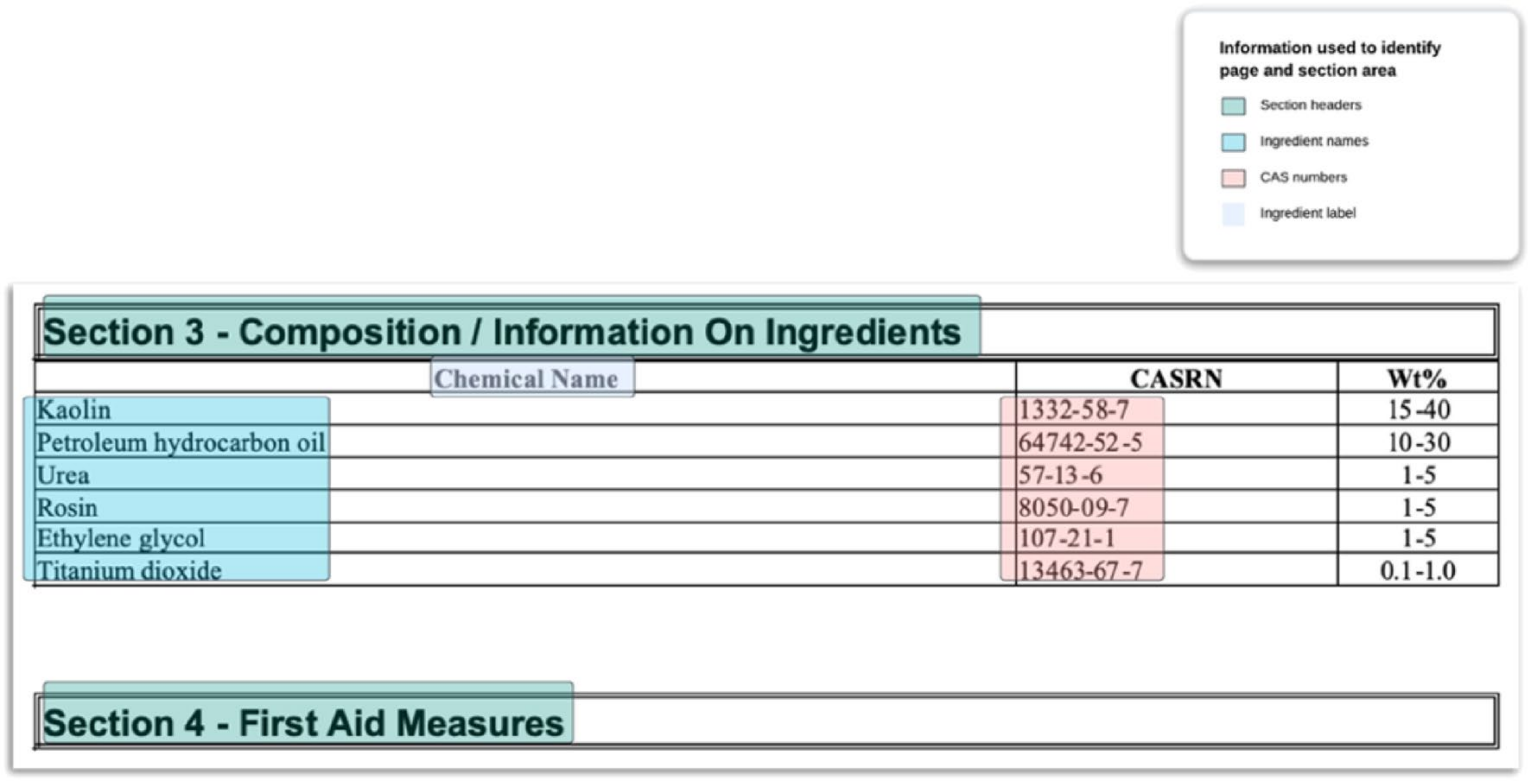
Cropped area of the composition section of an SDS document. Pieces of information used in the page selection and section detection processes of the pipeline are highlighted.

The purpose of this step was to identify the pages within the PDF file that contain the composition information. A combination of rules designed to identify the beginning and end of the composition section in the text, along with the presence of the relevant ingredient information within the section, was used. Regular expressions were used to identify the section headers. The regular expressions were designed to look for certain combinations of words generally present in the section headers. The ingredient information the system looked for at this step were the presence of CAS numbers using regular expressions^25^, the presence of ingredient labels using regular expressions, and the presence of ingredient names using a Named Entity Recognition (NER) model trained for this purpose (Fig. 3). NER is a task in NLP to identify key pieces of information (called entities) in a sentence or text. An entity can be a word or a group of words belonging to the same category. The model used was a pre-trained BERT model, finetuned on a custom dataset containing texts extracted from the composition section of the SDS documents (details in Supplementary Table S1). The pre-processed text was split into tokens and given as input (in chunks of size equal to the maximum sequence length of the model) to the BERT model and the output vector of the model was used for NER prediction of the ingredient names.

The model was designed to identify three categories of tokens: (1) the first word of the ingredient name, (2) the subsequent words of the ingredient name, and (3) the words not belonging to the ingredient name. In this step, the system used the model to look for the presence of any tokens belonging to an ingredient name. The following rules were used to identify the right page(s):

- Check if the beginning and end of the composition section is on the same page. If the text between the sections contains the relevant ingredient information, add the page to the list of correct pages.
- Check if only the beginning of the composition section is found on any page. If the text after this point contains the relevant ingredient information, add the page to the list of correct pages.
- Check if only the end of the ingredient section is found on any page. If the text before this point contains the relevant ingredient information, add the page to the list of correct pages.
- If no correct pages are identified, check if a CAS number is present in Section 1 of the document. If it is, then page 1 is the correct page.

The rules were also designed to handle cases where the table was split into multiple pages (Fig. 1), in which case, multiple correct pages were identified. This step restricts the search to the pages containing the appropriate section and improves the system’s overall precision.

### Image generation

To prepare the input for the next step, images of the SDS pages identified in the last step were generated using the pdf2 image^26^ library.

### Table/section detection

The purpose of this step was to identify the location of the table(s)/section area(s) containing the ingredient information in the image(s) generated in the previous step. Because in most cases, the ingredient information in an SDS is arranged in a tabular format, an object detection model was trained to identify the location of such tables from images (with and without the ingredient table) of the SDS document. The architecture of the model used was inspired by CascadeTabNet^27^, which combined the high-quality detective ability of Cascade Mask R-CNN^28^ with the high-resolution representations allowed by a pre-trained HRNet^29^ backbone network. The model was fine-tuned on a custom dataset containing images of SDS documents (details in Supplementary Table S1).

Cascade Mask R-CNN is a combination of the Cascade R-CNN^30^ architecture and a segmentation branch used for instance segmentation. The Cascade R-CNN architecture is a multi-stage extension of R-CNN^31^ designed to improve the quality of the detections using cascading techniques. While training an object detection model, the intersection over union threshold (IoU) is generally used to define positives and negatives, and the commonly used value for it is 0.5. This value can often lead to noisy detections, while increasing it frequently reduces the performance of the model due to overfitting and inference-time quality mismatch. To overcome these issues, the Cascade R-CNN architecture uses a sequence of detectors with an increasing IoU, where the output of one detector is used to train the next. The network is trained end-to-end, where each stage becomes increasingly better at discarding low-quality proposals of the previous stage, producing high quality detections at the final stage.

**Figure 4:**
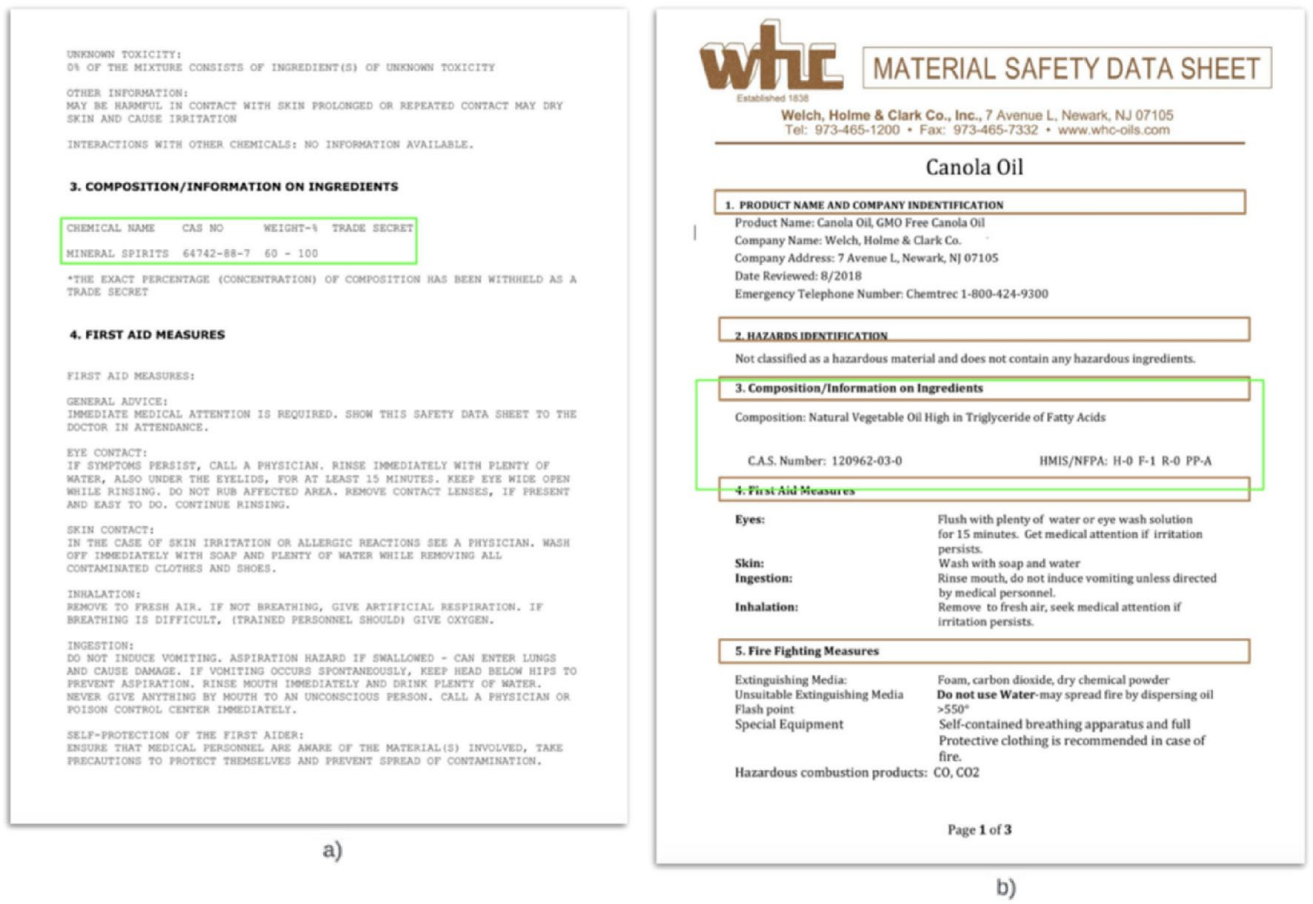
(**a**) Example of a borderless table detected (outlined). (**b**) Example of section detection for an SDS document where the composition information is not arranged in a table.

The output of the model was the coordinates of the table(s) containing the ingredient information in each page image. The model was trained to identify both bordered and borderless tables (Fig. 4a). To handle the cases where the model did not find a table, either because it failed to or because the information was not arranged in a tabular structure (Fig. 4b), a section detection step to identify the coordinates of the composition section was designed (to be used in the same way as the table coordinates in the subsequent steps). First, the open-source Optical Character Recognition (OCR) engine, Tesseract, was used to obtain the coordinates of every character in the right pages(s). Next, a combination of rules, similar to those used to identify the correct page, was used to identify the characters marking the beginning and end of the composition section in the recognized text. The coordinates of those characters were used as the coordinates of the composition section.

### Table data extraction.

Nowadays, SDS documents are easily available in digital form (PDF) and generally text-based, with a very small percentage being scanned documents (~ 4% in our dataset). Therefore, we avoided the use of image-based machine learning techniques for extracting the text from the identified area(s) of the documents, as they would have to rely on OCR, which would add a degree of error to the solution and are not required for a vast majority of the documents. We used Tabula^32^, a text-based table extraction software, which was able to handle a wide variety of table formats (using the Python wrapper tabula-py^33^). The table/section coordinates, the PDF document, and the relevant page numbers were given as input to the software to extract the text from the area. It returned the extracted text output in the form of a 2D data structure (pandas dataframe) retaining the tabular structure of the data, which was used in the subsequent step to identify the weight percentages and to obtain the association between the names, CAS numbers, and weight percentages.

### Ingredient information extraction

The purpose of this step was to remove the noise from the text extracted in the previous step and identify the ingredient names along with the corresponding CAS numbers and weight percentages. The first step of the process was identifying the ingredient names in the extracted text using the trained BERT NER model. The input to the model was the text of the table/area concatenated row-wise and split into tokens. Tokens to represent column and row separation—“[cs]” and “[rs]” were also inserted at the respective positions to help the model capture the layout information of the table (discussed more in Section “Results and discussion”). The model was designed to make a prediction for each token of the text individually (as described in Section “Page selection”) and additional post-processing rules were used to acquire the full name of the ingredients and to combine any rows wrongly split into multiple rows by the table extraction software. Next, the text of the table was scanned to look for these names and to identify the rows and columns containing them. The columns containing the CAS numbers and weight percentages were then identified using regular expressions, and the tabular structure of the data was used to get the association between the names, CAS numbers, and weight percentages. The regular expressions for CAS numbers were also designed to include non-numerical values like Trade Secret, Mixture, etc. The final output was a 2D structure with each row corresponding to an ingredient, and the columns corresponding to ingredient names, CAS numbers, and weight percentages. The weight percentages were split into 3 columns—upper, lower, and operator. Upper and lower represented the upper and lower limits of the range and the operator represented the relationship between the upper and lower values (e.g., if weight is “10–20”, upper is 20, lower is 10 and operator is “-”). Possible values for the operator were “-”, “ > ”, “ < ”, “ = ” (when the weight is an exact value), and “NaN”. This output can be stored in a structured database or in a file.

### Datasets

VelocityEHS has been performing manual indexing of SDS documents for its clients for decades and has a database of millions of indexed SDS documents. The format of the documents has undergone considerable changes over the years, and we decided to use all data available since 2019, consisting of more than 650,000 documents, for training and testing our models in this pipeline. In that dataset, we also had a list of documents that did not contain any composition/ingredient information; this list was used to train and test the no data classifier. In addition, we also used Amazon Web Services’ SageMaker’s Ground Truth tool, collaborating with a third-party annotation service provider, iMerit^34^, to obtain a number of additional labels needed to train and test our models:

- Bounding box annotations for the table containing the ingredient information for about 40,000 documents. These annotations were used to train and test the table detection model.
- Binary classification labels for about 10,000 documents with the labels indicating if a given page in the document contains any ingredient information or not. These labels were used to evaluate the rule-based page selection model.
- The manually indexed data had a significant degree of error, which was hindering our ability to accurately measure the performance of our pipeline. To overcome this issue, we used the annotation service to obtain the ingredient information (ingredient names, CAS numbers, and weight percentages) as text for about 20,000 documents, which were then used to evaluate the end-to-end pipeline.

## Results and discussion

Table 1 shows the performance of the different stages of the pipeline. For the no data classifier, the table shows the metrics for the class representing documents with no composition information (absolute numbers for both the classes are present in Supplementary Table S2). For the page selection stage, each page number correctly identified was counted as a true positive. For the NER model, the table shows the weighted average of three classes that the model was trained to identify. The weighted average accounts for the high degree of imbalance across the classes with ~ 90% of the tokens in the text not being part of the ingredient name. For the table detection model, an IoU threshold of 0.5 (commonly used) was used to define positives and negatives.

**Table 1:** Performance of various stages of the pipeline.

	Test dataset	Precision	Recall
No data classifier	Mentioned in Section “Datasets”.c	0.93	0.84
Page selection	Mentioned in Section “Datasets”.b	0.96	0.97
NER model	Test split mentioned in Supplementary Table S1	0.99*	0.99*
Table detection	Test split mentioned in Supplementary Table S1 (and Section. “Datasets”.a)	0.94	0.94

*Weighted average.

For measuring the performance of the end-to-end pipeline, three types of metrics were defined (for documents containing composition information):

1. Tier 1—For each of the entities, ingredient name, CAS number, upper, lower, and operator, individually.
2. Tier 2—For all values corresponding to an ingredient combined.
3. Tier 3—For all values of all ingredients in an SDS combined.

Table 2 contains the metrics obtained for each tier. It also contains the combined metrics obtained for all documents where the results for documents with and without composition information (Table 1) were combined (absolute numbers for calculating the precision and recall values in Table 2 are present in Supplementary Table S2). There is a trade-off between precision and recall at each step of the pipeline, and the metrics show the results of a version of the system designed to maximize precision at the cost of some loss in recall.

**Table 2:** Performance of the end-to-end pipeline on the dataset mentioned in Section “Datasets”.c.

	Precision	Recall
Documents with data		
Ingredient	0.991	0.697
CAS	0.998	0.704
Operator	0.981	0.734
Upper	0.989	0.724
Lower	0.993	0.73
Tier 2 (ingredient level)	0.974	0.675
** Tier 3 (Document level)**	**0.93**	**0.701**
** All documents**	**0.931**	**0.713**

Significant values are in bold.

Each stage of the pipeline improved the overall performance of the system by systematically narrowing down the region of search. For example, the no data filter removed documents that do not contain any composition information but contained pieces of text that may seem like ingredient names to an NER model (for e.g., non-hazardous chemicals). Although it seems that an alternative to having the filter could be to just use the pipeline to determine if the document has any composition information, the issue with that approach is that the pipeline may not make any prediction due to failures at any one of the stages, rather than the document simply not containing any composition information. It is easier for an ML system to learn whether a document has any composition information or not than to actually find it. This is also apparent from the fact that the metrics for the no data classifier are higher than the recall of the end-to-end pipeline.

This concept is also true for each of the other stages of the pipeline. In fact, the intuition behind the effectiveness of the pipeline is that the problem of extracting the composition information is broken down into smaller easier steps. The metrics for each of those steps are high enough (Table 1) to lead to a high end-to-end performance of the system. The page selection step prevented the table detection model from finding other tables containing a list of ingredients (without weight percentages) in other sections of the document. The table/area detection step not only kept the layout of the information intact, but also reduced the area of search for ingredient names. This was validated by experiments where the entire text of the document was used to train and identify ingredient names using an NER model, as opposed to just the text of the tables/areas; the metrics for the latter were significantly better.

After getting the text of the table/area, the next step of the ingredient information extraction process was to find the ingredient names using the BERT NER model. Although this step may benefit from the use of a multimodal technique, the transformer-based architecture of the BERT model seemed to be able capture both the textual context and the layout information in the extracted text. For example, CAS numbers, usually present next to the ingredient names in the extracted text, provided important textual context to help the model identify the ingredient names, and the performance of the model degraded when they were removed from the text. The text from the table was concatenated row-wise and fed to the NER model as the input where empty cells were represented by “NA”. This meant that in most cases, because the ingredient names were in the same column, each ingredient name was present in the extracted text roughly after the same interval of tokens. This ensured that the layout information of the table was still retained in the extracted text to some extent. The performance of the model degraded when the “NA”s were removed, showing that the model was sensitive to this information. The performance of the model also degraded when the text was extracted column-wise instead of row-wise, and when the tokens representing column and row separation were not added to the input to the model.

## Conclusion and future work

In this paper, we presented an automated system for extracting and indexing composition information from SDS documents using machine learning techniques. We illustrated the benefits of an ensemble multi-layered technique, which uses the format of an SDS document to narrow down the area of interest at each layer while breaking down the problem into simpler steps, and showed the effectiveness of the technique on our datasets. This technique can also be extended for extracting other pieces of information from SDS documents. With SDSs being essential for numerous practices within the EHS and ESG domains, such a system is highly valuable for several industries.

Although the system uses both textual and visual information in various stages of the pipeline separately, each of the stages can benefit from the use of multimodal techniques. For example, the table detection step could benefit from textual information about the section headers, as well as the contents of the table. The NER model was able to capture some of the layout information while identifying ingredient names, but error analysis of the model revealed it failed to do so on several occasions. The table data extraction software failed to identify the structure of the table correctly on several occasions, especially when the contents of the cell spanned multiple lines, or the columns were too close to each other with no borders between them. A more robust table structure recognition model, combined with a high-quality OCR model, can significantly improve the performance of the system.

### Supplementary Information

Supplementary Tables.

## Data Availability

The data used to train and test our models are available from VelocityEHS but restrictions apply to the availability of these data, which were obtained through labor intensive expensive processes, and are proprietary, and so are not publicly available. Data are however available from the authors upon reasonable request and with permission of VelocityEHS.

## Code availability

The code for reproducing the designed system is available from VelocityEHS but restrictions apply to the availability of the code, which is proprietary, and so is not publicly available. The code is however available from the authors upon reasonable request and with permission of VelocityEHS.
