# Supplementary material for: A machine learning driven automated system for safety data sheet indexing
Source: Sci Rep. 2024 Feb 22;14:4415. doi: 10.1038/s41598-024-55231-1 (PMC10883951; doi:10.1038/s41598-024-55231-1)
Supplement: Supplementary file 1 — Supplementary Tables. [file 41598_2024_55231_MOESM1_ESM.docx]

**A Machine Learning Driven Automated System for Safety Data Sheet Indexing**

Aatish Suman*^1^, Misbah Khan^1^, Veeru Talreja^1^, Julia Penfield*^1^, Stephanie Crowell^1^

^1^Velocity EHS Inc., Chicago, IL 60654 (US)

*[asuman@ehs.com](mailto:asuman@ehs.com); [dr.juliapenfield@ieee.org](mailto:dr.juliapenfield@ieee.org)

**Supplementary Table S1 | Details of models trained for the pipeline**

|  | **No data classifier** | **NER model** | **Table detection model** |
| --- | --- | --- | --- |
| **Architecture** | pre-trained BERT | pre-trained BERT | Cascade Mask R-CNN; pre-trained HRNet backbone |
| **Dataset** | 60k documents (text); manually indexed dataset; 90/10 train/test split | 243k documents (text from table/section area); manually indexed dataset; 97.5/2.5 train/test split | 40k images (with and without right table); custom annotated dataset; 85/15 train/test split |
| **Batch size** | train – 32; test – 8 | train – 32; test – 8 | train & test – 4 |
| **Epochs** | 5 | 8 | 17 |
| **Maximum sequence length** | 512 | 512 | N/A |
| **Image size & resolution** | N/A | N/A | original – 1700 x 2200; resized – 1333 x 800; DPI – 200 |
| **Inference confidence threshold** | N/A | N/A | 0.85 |

**Supplementary Table S2 | Absolute numbers used for calculating precision and recall in Tables 1 and 2**

| \| **No data classifier (in Table 1)** \| \| \| \| \| --- \| --- \| --- \| --- \| \|  \| Has no data predicted \| Has data predicted \| **Recall** \| \| Has no data (6%) \| 951 \| 188 \| **0.835** \| \| Has data (94%) \| 75 \| 17932 \|  \| \| **Precision** \| **0.927** \|  \|  \| | \| **Ingredient (in Table 2)** \| \| \| \| \| --- \| --- \| --- \| --- \| \|  \| Ingredients predicted \| Ingredients not predicted \| **Recall** \| \| Ingredients present \| 46707 \| 20319 \| **0.697** \| \| Ingredients not present \| 430 \| 0 \|  \| \| **Precision** \| **0.991** \|  \|  \| |
| --- | --- | --- | --- | --- | --- | --- | --- | --- | --- | --- | --- | --- | --- | --- | --- | --- | --- | --- | --- | --- | --- | --- | --- | --- | --- | --- | --- | --- | --- | --- | --- | --- | --- | --- | --- | --- | --- | --- | --- | --- | --- |

| \| **CAS (in Table 2)** \| \| \| \| \| --- \| --- \| --- \| --- \| \|  \| CAS predicted \| CAS not predicted \| **Recall** \| \| CAS present \| 46835 \| 9935 \| **0.825** \| \| CAS not present \| 94 \| 0 \|  \| \| **Precision** \| **0.998** \|  \|  \| | \| **Operator (in Table 2)** \| \| \| \| \| --- \| --- \| --- \| --- \| \|  \| Operators predicted \| Operators not predicted \| **Recall** \| \| Operators present \| 46446 \| 16746 \| **0.735** \| \| Operators not present \| 900 \| 0 \|  \| \| **Precision** \| **0.981** \|  \|  \| |
| --- | --- | --- | --- | --- | --- | --- | --- | --- | --- | --- | --- | --- | --- | --- | --- | --- | --- | --- | --- | --- | --- | --- | --- | --- | --- | --- | --- | --- | --- | --- | --- | --- | --- | --- | --- | --- | --- | --- | --- | --- | --- |

| \| **Upper (in Table 2)** \| \| \| \| \| --- \| --- \| --- \| --- \| \|  \| Uppers predicted \| Uppers not predicted \| **Recall** \| \| Uppers present \| 46670 \| 17791 \| **0.724** \| \| Uppers not present \| 519 \| 0 \|  \| \| **Precision** \| **0.989** \|  \|  \| | \| **Lower (in Table 2)** \| \| \| \| \| --- \| --- \| --- \| --- \| \|  \| Lowers predicted \| Lowers not predicted \| **Recall** \| \| Lowers present \| 46690 \| 17269 \| **0.73** \| \| Lowers not present \| 329 \| 0 \|  \| \| **Precision** \| **0.993** \|  \|  \| |
| --- | --- | --- | --- | --- | --- | --- | --- | --- | --- | --- | --- | --- | --- | --- | --- | --- | --- | --- | --- | --- | --- | --- | --- | --- | --- | --- | --- | --- | --- | --- | --- | --- | --- | --- | --- | --- | --- | --- | --- | --- | --- |

| \| **Tier 2 (in Table 2)** \| \| \| \| \| --- \| --- \| --- \| --- \| \|  \| Tier 2 (predicted) \| Tier 2 (not predicted) \| **Recall** \| \| Tier 2 (present) \| 46071 \| 22185 \| **0.675** \| \| Tier 2 (not present) \| 1250 \| 0 \|  \| \| **Precision** \| **0.974** \|  \|  \| | \| **Tier 3 (in Table 2)** \| \| \| \| \| --- \| --- \| --- \| --- \| \|  \| Tier 3 (predicted) \| Tier 3 (not predicted) \| **Recall** \| \| Tier 3 (present) \| 12695 \| 5425 \| **0.701** \| \| Tier 3 (not present) \| 962 \| 0 \|  \| \| **Precision** \| **0.93** \|  \|  \| |
| --- | --- | --- | --- | --- | --- | --- | --- | --- | --- | --- | --- | --- | --- | --- | --- | --- | --- | --- | --- | --- | --- | --- | --- | --- | --- | --- | --- | --- | --- | --- | --- | --- | --- | --- | --- | --- | --- | --- | --- | --- | --- |

| **All documents (in Table 2)** | | | |
| --- | --- | --- | --- |
|  | All documents (predicted) | All documents (not predicted) | **Recall** |
| All documents (present) | 13646 | 5500 | **0.713** |
| All documents (not present) | 1009 | 0 |  |
| **Precision** | **0.931** |  |  |
